# Separate F-Type Plasmids Have Shaped the Evolution of the *H*30 Subclone of *Escherichia coli* Sequence Type 131

**DOI:** 10.1128/mSphere.00121-16

**Published:** 2016-06-29

**Authors:** Timothy J. Johnson, Jessica L. Danzeisen, Bonnie Youmans, Kyle Case, Katharine Llop, Jeannette Munoz-Aguayo, Cristian Flores-Figueroa, Maliha Aziz, Nicole Stoesser, Evgeni Sokurenko, Lance B. Price, James R. Johnson

**Affiliations:** a Department of Veterinary and Biomedical Sciences, University of Minnesota, St. Paul, Minnesota, USA; b Division of Pathogen Genomics, Translational Genomics Research Institute, Flagstaff, Arizona, USA; c Department of Occupational and Environmental Health, George Washington University, Washington, DC, USA; d Modernising Medical Microbiology Consortium, Nuffield Department of Medicine, John Radcliffe Hospital, University of Oxford, Oxford, United Kingdom; e Department of Microbiology, University of Washington, Seattle, Washington, USA; f Minneapolis Veterans Health Care System, Minneapolis, Minnesota, USA; g Department of Medicine, University of Minnesota, Minneapolis, Minnesota, USA; JMI Laboratories

**Keywords:** *Escherichia coli*, ST131, genomes, plasmids

## Abstract

The extraintestinal pathogenic *Escherichia coli* (ExPEC) *H*30 subclone of sequence type 131 (ST131-*H*30) has emerged abruptly as a dominant lineage of ExPEC responsible for human disease. The ST131-*H*30 lineage has been well described phylogenetically, yet its plasmid complement is not fully understood. Here, single-molecule, real-time sequencing was used to generate the complete plasmid sequences of ST131-*H*30 isolates and those belonging to other ST131 clades. Comparative analyses revealed separate F-type plasmids that have shaped the evolution of the main fluoroquinolone-resistant ST131-*H*30 clades. Specifically, an F1:A2:B20 plasmid is strongly associated with the *H*30R/C1 clade, whereas an F2:A1:B− plasmid is associated with the *H*30Rx/C2 clade. A series of plasmid gene losses, gains, and rearrangements involving IS*26* likely led to the current plasmid complements within each ST131-*H*30 sublineage, which contain several overlapping gene clusters with putative functions in virulence and fitness, suggesting plasmid-mediated convergent evolution. Evidence suggests that the *H*30Rx/C2-associated F2:A1:B− plasmid type was present in strains ancestral to the acquisition of fluoroquinolone resistance and prior to the introduction of a multidrug resistance-encoding gene cassette harboring *bla*_CTX-M-15_. *In vitro* experiments indicated a host strain-independent low frequency of plasmid transfer, differential levels of plasmid stability even between closely related ST131-*H*30 strains, and possible epistasis for carriage of these plasmids within the *H*30R/Rx lineages.

**IMPORTANCE** A clonal lineage of *Escherichia coli* known as ST131 has emerged as a dominating strain type causing extraintestinal infections in humans. The evolutionary history of ST131 *E. coli* is now well understood. However, the role of plasmids in ST131’s evolutionary history is poorly defined. This study utilized real-time, single-molecule sequencing to compare plasmids from various current and historical lineages of ST131. From this work, it was determined that a series of plasmid gains, losses, and recombinational events has led to the currently circulating plasmids of ST131 strains. These plasmids appear to have evolved to acquire similar gene clusters on multiple occasions, suggesting possible plasmid-mediated convergent evolution leading to evolutionary success. These plasmids also appear to be better suited to exist in specific strains of ST131 due to coadaptive mutations. Overall, a series of events has enabled the evolution of ST131 plasmids, possibly contributing to the lineage’s success.

## Abstract (precis)

A clonal lineage of *Escherichia coli* known as ST131 has emerged as a dominating strain type causing extraintestinal infections in humans. The evolutionary history of ST131 *E. coli* is now well understood. However, the role of plasmids in ST131’s evolutionary history is poorly defined. This study utilized real-time, single-molecule sequencing to compare plasmids from various current and historical lineages of ST131. From this work, it was determined that a series of plasmid gains, losses, and recombinational events has led to the currently circulating plasmids of ST131 strains. These plasmids appear to have evolved to acquire similar gene clusters on multiple occasions, suggesting possible plasmid-mediated convergent evolution leading to evolutionary success. These plasmids also appear to be better suited to exist in specific strains of ST131 due to coadaptive mutations. Overall, a series of events has enabled the evolution of ST131 plasmids, possibly contributing to the lineage’s success.

## INTRODUCTION

**FIG 1:**
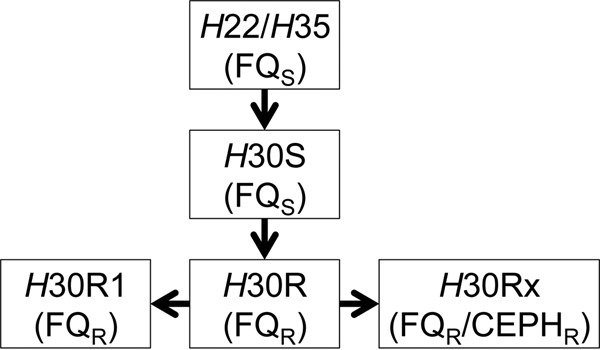
ST131 subclade emergence and designations, based upon *fimH* allele. FQ_S_, fluoroquinolone sensitive; FQ_R_, fluoroquinolone resistant; CEPH_R_, cephalosporin resistant.

A major global shift has occurred in the distribution of the extraintestinal pathogenic *Escherichia coli* (ExPEC) strains that cause infections of the urinary tract and other extraintestinal sites (1). This shift is best described as an emergence and dominance of *E. coli* sequence type 131 (ST131), a clonal lineage of ExPEC not previously recognized among clinical isolates (2). Within ST131, it is well established that different sublineages have emerged. These sublineages are designated according to their characteristic *fimH* allele (type 1 fimbriae adhesin gene), phylogenetic clade (A, B, C1, and C2, as recently proposed), and resistance profile (3, 4). The most clinically important and expanded of the ST131 sublineages are *H*30R1 (where “R” indicates resistance to fluoroquinolones), or clade C1, which has acquired fluoroquinolone resistance; and *H*30Rx, or clade C2, which frequently carries *bla*_CTX-M-15_, conferring extended-spectrum β-lactam resistance (5). *H*30R1/C1 and *H*30Rx/C2 are sister clades that appear to have evolved from a common ancestor designated *H*30R. The predecessor to *H*30R was *H*30S (where “S” indicates susceptibility to fluoroquinolones), which is thought to have evolved from *H*22/B, a fluoroquinolone- and cephalosporin-susceptible ancestor (Fig. 1) (6, 7). It has been proposed that the *H*30Rx/C2 clade emerged in part through acquisition of a plasmid containing *bla*_CTX-M-15_ and that this element has subsequently integrated into different chromosomal sites in some strains (5, 6). For simplicity, *fimH* allele designations for the ST131 clonal subsets will be used throughout.

While there is abundant information regarding the clinical aspects and phylogenetics of ST131, the plasmids of ST131 are diverse and not fully defined. F-type plasmids containing multiple plasmid replicons have been identified as dominant within ST131 overall, but their relationships and differential evolution between the various ST131 sublineages are poorly understood (8, 9). The most comprehensive analysis of ST131 plasmids to date (10), which utilized draft genomic sequences, showed that *H*30Rx strains commonly contain an F plasmid with a plasmid multilocus sequence type (pMLST) of F2:A1:B−, based upon the 9 strains examined. It is currently unknown what advantages these plasmids confer besides resistance to antibiotics, although it seems likely that ST131 strains have adapted over time to more efficiently harbor these plasmids (11).

Here, we sought to clarify the within-clade evolution of ST131 in relation to its plasmids by performing comparative plasmid sequencing and analysis of completed plasmids from across the ST131 phylogeny. We also sought to clarify experimentally the *in vitro* transfer potential and stability of different ST131 plasmids.

## RESULTS AND DISCUSSION

### Different F-type plasmids define ST131 sublineages.

**FIG 2:**
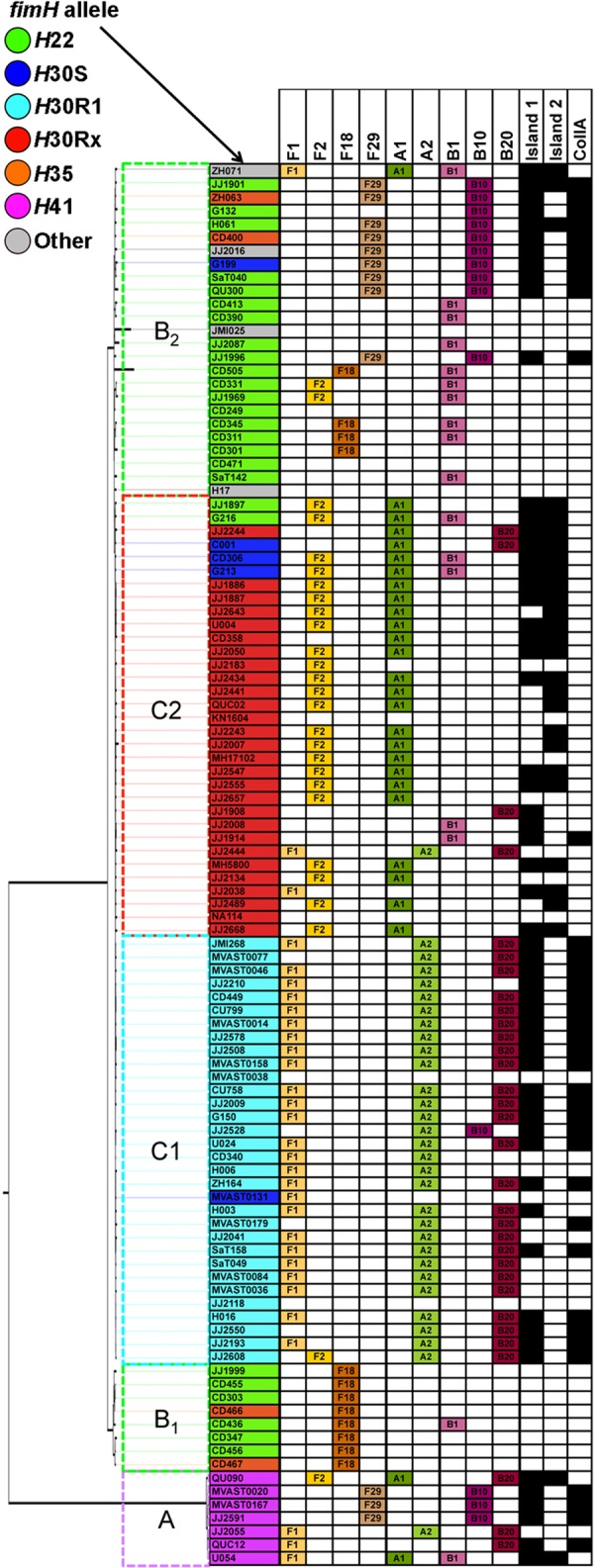
Distribution of F plasmid allele types, island 1, island 2, and the ColIa-containing island among ST131 strains. Strains are ordered by their position in the ST131 phylogenetic tree adapted from reference 5 and colored by *fimH* allele type according to the figure legend. Strain clades (A, B_1_, B_2_, C1, and C2) are designated by colored and labeled dashed boxes. Under island 1, island 2, or ColIa, a black-shaded box indicates >99% nucleotide similarity across >90% of the sequence queried.

A previous collection of 104 diverse ST131 strains described by Price et al. (5) was used first to assess possession of F plasmid allele types by a multilocus analysis approach (Fig. 2). This approach assessed alleles of the FIIA (F), FIA (A), and FIB (B) replicons. Of the 27 *H*30Rx strains, 19 (70%) possessed the F2 allele, 20 (74%) possessed the A1 allele, and 18 (67%) possessed the F2:A1 combination. This was significantly higher (*P* < 0.0001) than the prevalence of F2:A1 plasmids among the 32 *H*30R1 strains (0%). In contrast, 25 of the 32 *H*30R1 strains (78%) possessed the F1 allele, 30 (94%) possessed the A2 allele, and 26 (81%) possessed the B20 allele. Thus, the F1:A2:B20 allele combination was fully present in 22 (69%) of the *H*30R1 strains, versus only 1 (4%) of the *H*30Rx strains (*P* < 0.0001).

Although F2:A1 (dominating among *H*30Rx isolates) and F1:A2:B20 (dominating among *H*30R1 isolates) were not dominant among strains considered ancestral or basal to *H*30R1 and *H*30Rx, they nonetheless were identified in several such strains. For example, the F2:A1 allele combination also occurred among *H*22 and *H*41 strains, and the F1:A2:B20 allele occurred among *H*30Rx and *H*41 strains. However, the more common replicon alleles among ancestral strains were F18, F29, B1, and B10. Specifically, the F29:B10 allele combination was found among *H*41 and *H*22, H30S, and *H*35 strains in the upper portion of the ST131 tree (clade B_2_) and was absent from *H*30R1 and *H*30Rx strains (Fig. 2). In contrast, the F18 allele was found among basal *H*22 and *H*35 strains in the bottom portion of the ST131 tree (clade B_1_) and was absent from *H*41, *H*30R1, and *H*30Rx strains. Thus, although plasmid allele combinations correlate strongly with strain phylogenetic background, in exceptional instances the same combinations occur in a small number of ancestral isolates. Overall, these data are consistent with acquisition and subsequent loss of F18 and F29:B10 plasmid types during the evolution of ST131, prior to the emergence of ST131-*H*30.

### ST131-*H*30Rx strains contain an F2:A1 plasmid similar to plasmids from closely related *H*22 and *H*30S strains.

**FIG 3:**
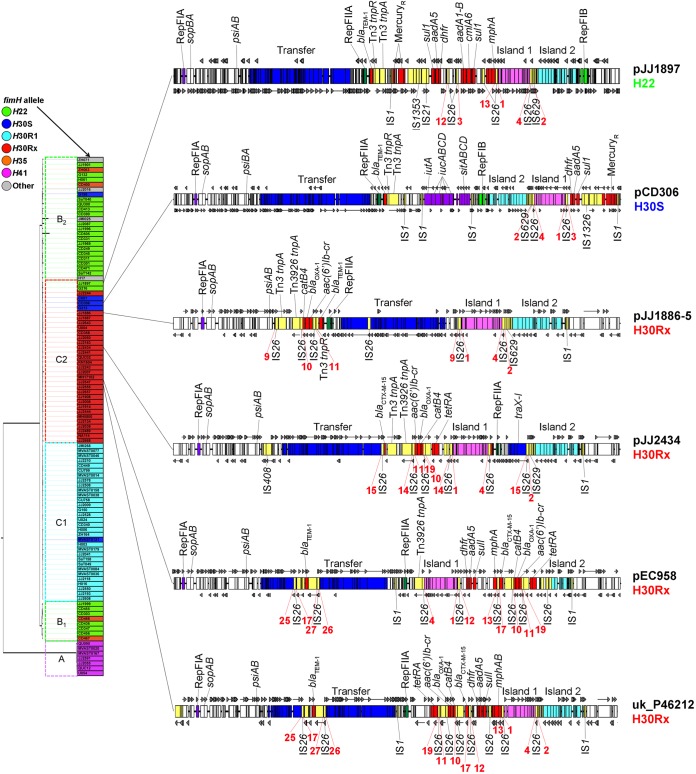
Linear maps of completed F2:A1 plasmids from *H*22, *H*30S, and *H*30Rx strains. Maps are not to scale. Lines from each map indicate the locations of corresponding isolates in the ST131 phylogenetic tree, with plasmid names colored by *fimH* allele type. Red numbering below each plasmid indicates IS*26* tracer sequences, with those of the same number having the same 8-bp flanking sequence.

The completed sequences of four F2:A1 plasmids from ST131-*H*30Rx strains, one F2:A1:B23 plasmid from *H*22, and one F2:A1:B1 plasmid from *H*30S were examined for their basic structure and orientation of key loci (Table 1; Fig. 3) (6, 12–20). Regardless of host strain clonal background, all six sequenced plasmids contained their expected replicons, stability and maintenance genes *psiAB* and *sopAB*, and a conjugative transfer region. They all also possessed two genetic loci that were labeled here as “island 1” and “island 2” (see Fig. S1 in the supplemental material). Island 1 contained nine open reading frames, including a predicted ABC-type transport system associated with iron transport and a putative DNA-binding transcriptional regulator. Island 2 contained 12 open reading frames, including a predicted system for carbohydrate transport, another for carbohydrate modification, and a predicted helix-turn-helix (HTH)-type transcriptional regulator.

10.1128/mSphere.00121-16.1 Figure S1  Genes found within genetic regions labeled “Island 1” and “Island 2.” Genes are colored by system and labeled by their predicted protein function. Download Figure S1, TIF file, 0.2 MB. Copyright © 2016 Johnson et al. 2016 Johnson et al. This content is distributed under the terms of the Creative Commons Attribution 4.0 International license.

**TABLE 1:** Isolates and plasmids analyzed in this study

Strain	*fimH* allele	Molecule	Size (bp)	Accession no.	Reference
SaT040	*H*22	Chromosome	5,061,821	CP014495	This study
		pSaT040	114,223	CP014496	This study
JJ1897	*H*22	Chromosome	5,174,541	CP013837	This study
		pJJ1897_1	140,502	CP013836	This study
G749	*H*22	Chromosome	4,897,758	CP014488	This study
		pG749_1	149,732	CP014489	This study
		pG749_2	109,910	CP014490	This study
		pG749_3	62,572	CP014491	This study
CD306	*H*30S	Chromosome	5,073,822	CP013831	This study
		pCD306	145,221	CP013832	This study
G199	*H*30S	Chromosome	NA^a^	LSUP00000000	This study
		pG199_1	114,233	LSUP00000000	This study
G150	*H*30R1	Chromosome	NA	LQHK00000000	This study
		pG150	137,382	LQHK00000000	This study
JJ2434	*H*30Rx	Chromosome	5,128,614	CP013835	This study
		pJJ2434_1	126,302	CP013833	This study
		pJJ2434_2	62,183	CP013834	This study
ZH063	*H*35	Chromosome	5,033,359	CP014522	This study
		pZH063_1	114,223	CP014523	This study
		pZH063_2	49,467	CP014524	This study
MVAST0167	*H*41	Chromosome	4,806,946	CP014492	This study
		pMVAST0167_1	128,305	CP014493	This study
		pMVAST0167_2	78,992	CP014494	This study
MNCRE44	*H*30R1	pMNCRE44_6	122,966	CP010882	12
JJ1886	*H*30Rx	pJJ1886-5	110,040	CP006789	13
JJ1887	*H*30Rx	pJJ1887-4	107,507	CP014321	20
uk_P46212	*H*30Rx	uk_P46212	143,748	CP013657	6
L46	*H*30Rx	pEC_L46	144,871	GU371929	14
A	*H*30Rx	pEK499	117,536	EU935739	15
EC958	*H*30Rx	pEC958	135,602	HG941719	16
UTI89	ND^b^	pUTI89	114,230	CP000244	17
Uncultured DNA^c^	ND	pRSB225	164,550	JX127248	18
UMN026	ND	p1ESCUM	122,301	CU928148	19

a NA, draft sequence where chromosome is in multiple contigs.

b ND, not determined.

c The plasmid was not isolated; it was transformed from wastewater sludge plasmid DNA into *E. coli*.

While these sequenced F2:A1-containing plasmids were generally similar in overall structure, some key differences existed between those from *H*30Rx strains compared with those from *H*22 and *H*30S strains. Specifically (i) only those F2:A1 plasmids from *H*22 and *H*30S strains contained an FIB replicon (B23 and B1, respectively), (ii) islands 1 and 2 were inverted in the F2:A1-containing plasmids from *H*22 and *H*30S strains, compared to those from *H*30Rx strains, and (iii) all of the F2:A1 plasmids from *H*30Rx strains sequenced here, compared to none from the *H*22 or *H*30S strains, included a resistance locus containing *aac(6′)Ib-cr*, *bla*_OXA-1_, and *catB4*. Notably, numerous copies of IS*26* were identified throughout these F2:A1 plasmids, regardless of clonal background. IS*26* elements were associated with many rearrangements (discussed below).

**FIG 4:**
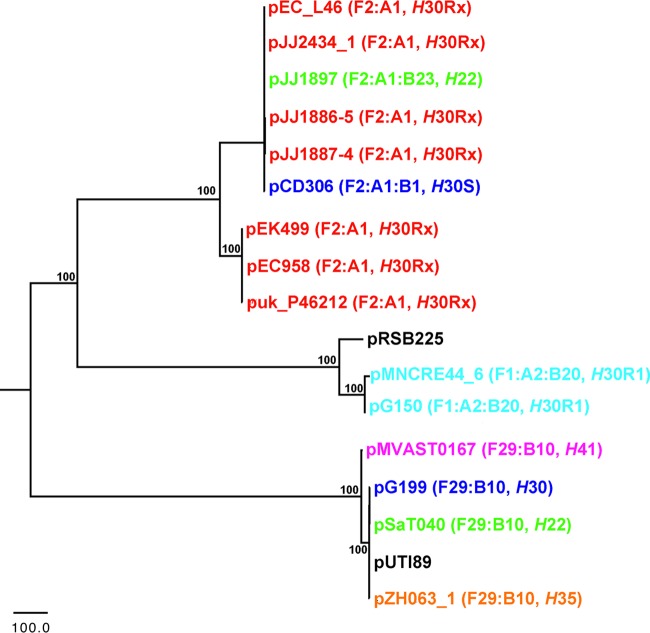
Inferred phylogenetic relationships of sequenced ST131 plasmids and closely related database plasmids. Evolutionary history was inferred by the maximum parsimony method, based on 2,414 shared SNP positions among the core regions (32,678 bp) of all plasmids analyzed. Repetitive elements were excluded from the analysis. Isolates are colored by the ST131 sublineage noted in the labels. Numbers at nodes represent bootstrap values based upon 1,000 replicates.

Using similarity searches of draft genome sequences, the presence of islands 1 and 2 was sought among all study isolates that contained an F2:A1 plasmid type. Within this subset (*n* = 24), 15 isolates (63%) contained island 1 and 21 (88%) contained island 2. Islands 1 and 2 were also present uniformly among those *H*22 and *H*30S isolates (which are considered basal to the *H*30Rx clade) that exhibited the F2:A1 allelic combination. Whole-plasmid alignments were performed, and phylogenetic inferences were made using conserved single nucleotide polymorphisms (SNPs) in nonrepetitive regions (Fig. 4). This demonstrated that pJJ1897 (*H*22) and pCD306 (*H*30S) fell within a monophyletic clade that also included sequenced *H*30Rx F2:A1 plasmids. Collectively, these results support acquisition of this plasmid type by ST131-*H*22 prior to the emergence of *H*30R1 and *H*30Rx.

### ST131-*H*30R1 strains contain an F1:A2:B20 plasmid distinct from the plasmids of *H*30Rx and closely related ST131 strains.

**FIG 5:**
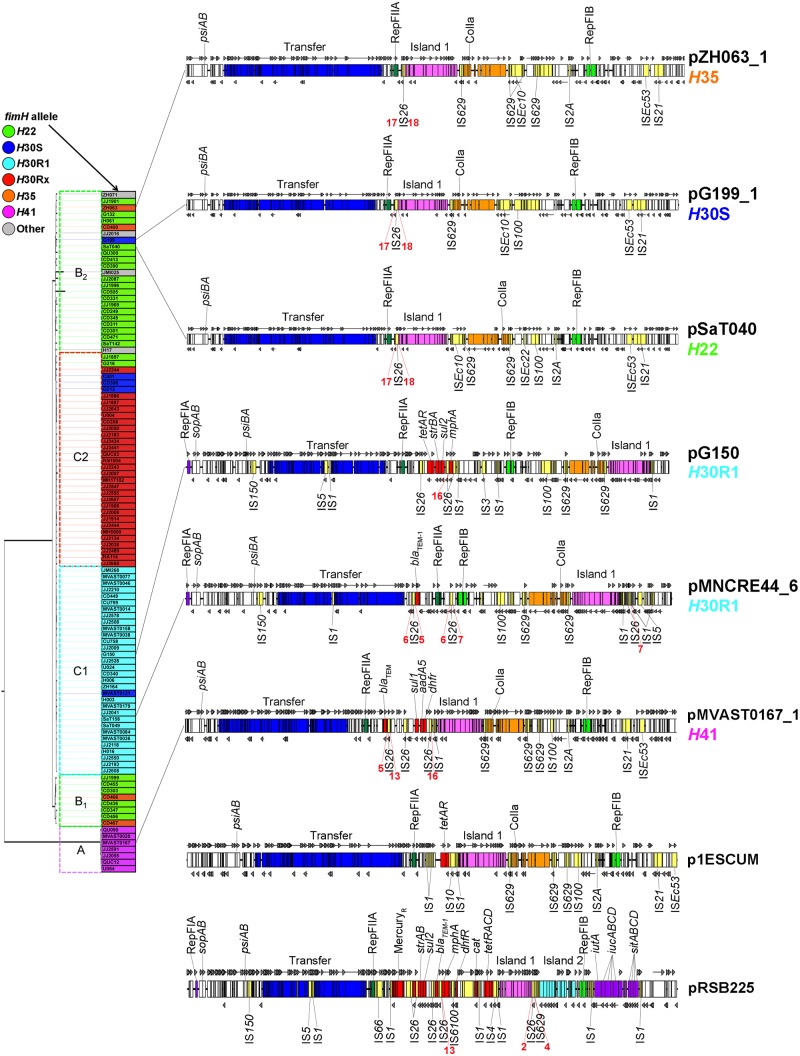
Linear maps of completed ColIa-containing plasmids from *H*22, *H*30S, *H*35, *H*41, and *H*30R1 isolates. Maps are not to scale. Lines from each map indicate the location of the corresponding isolate in the ST131 phylogenetic tree, with plasmid names colored by *fimH* allele type. Red numbering below each plasmid indicates IS*26* tracer sequences.

A second F-type plasmid (F1:A2:B20) was identified among *H*30R1 strains, distinct from the F2:A1 plasmid identified in *H*30Rx strains (Fig. 5). Key features of the sequenced F1:A2:B20 plasmids included possession of their expected FIA-FIIA-FIB replicons, plasmid stability genes *sopAB* and *psiAB*, and island 1. An additional feature found in all of these plasmids, but lacking in *H*30Rx plasmids, was a 10-kb region containing the colicin ColIa immunity-encoding gene, *cjrABC*, and the enterotoxin-encoding *senB* gene (designated ColIa in Fig. 5) (21). Of the 32 *H*30R1 strains, 23 (72%) contained both island 1 and the ColIa-containing region. Another differentiating feature of the F1:A2:B20 plasmids of *H*30R1, compared to the F2:A1 plasmids of *H*30Rx, was that they lacked island 2. Based on core plasmid SNPs, the F2:A1:B20 plasmids of *H*30R1 were clearly distinct from the F2:A1 plasmids of *H*30Rx, differing by greater than 1,000 core SNPs (Fig. 4).

A third plasmid type (F29:B10) was identified among non-*H*30R1 ST131 strains, including pZH063_1 (*H*35), pG199_1 (*H*30S), pSaT040 (*H*22), and pMVAST0167_1 (*H*41). These plasmids were highly similar to one another and similar in structure to the F1:A2:B20 plasmids of *H*30R1 (Fig. 5). The main notable gross differences between the F29:B10 and F1:A2:B20 plasmid types were that the FIA replicon and *sopAB* were absent from the F29:B10 plasmids and the ColIa-containing and island 1 regions were in reverse orientation and at different locations on the F29:B10 plasmids. Phylogenetically, however, F29:B10 and F1:A2:B20 plasmids were clearly distinct from one another, thus indicating that at least three plasmid types (F2:A1, F1:A2:B20, and F29:B10) have been introduced into the ST131 clade and harbor some overlapping traits (Fig. 4).

To determine possible origins for the plasmid types, we compared sequenced ST131 plasmids with those in the NCBI reference database (Fig. 4 and 5). Two closely related plasmids were identified harboring similar traits: p1ESCUM from an ExPEC strain (GenBank accession no. CU928148) and pRSB225 from uncultured wastewater treatment sludge (GenBank accession no. JX127248). Interestingly, the F1:A2:B20 plasmids from *H*30R1 strains were phylogenetically most similar to pRSB225, which also contained island 2, the aerobactin siderophore system (22), and the Sit iron transport system (23). In contrast, the other ColIa-containing plasmids (pZH063_1, pG199_1, pSaT040, and pMVAST0167_1) were phylogenetically most similar to p1ESCUM. This suggests that a pRSB225-like plasmid may have been introduced initially into ST131, serving as a reservoir for aerobactin, Sit, and islands 1 and 2. However, phylogenetic analyses of regions containing island 1 and ColIa (data not shown) from sequenced ST131 plasmids demonstrate multiple discrete clades. This suggests multiple introductions of these mobile regions, not a single introduction, supporting the idea that they were brought in multiple times via different plasmid types.

**FIG 6:**
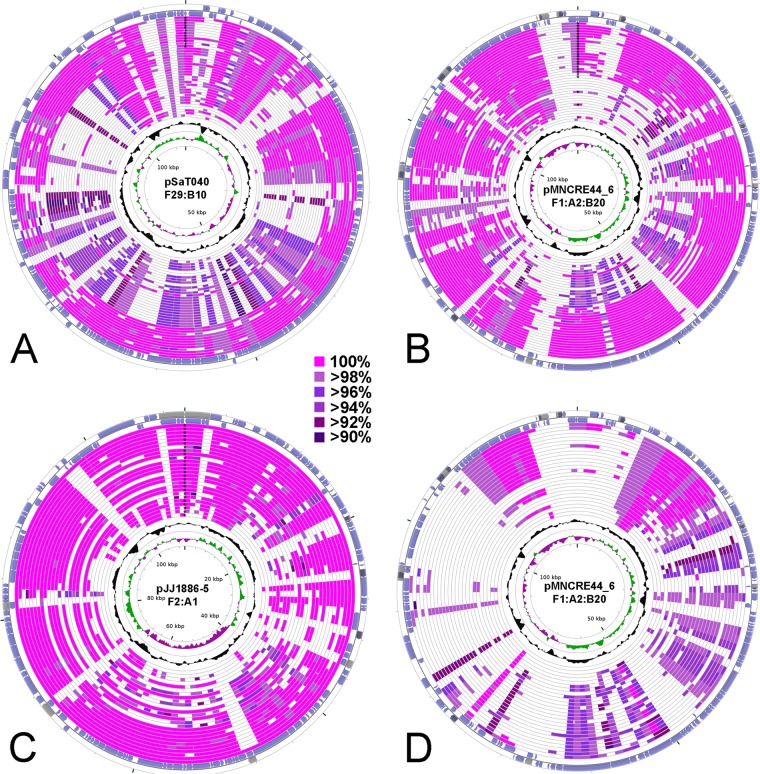
Circular maps displaying nucleotide conservation of plasmid types across ST131 sublineages. (A) pSaT040 (F29:B10 [accession no. CP014496]) used as reference compared to draft assemblies of ColIa-positive isolates. The outer 12 rings are F29:B10-positive isolates, and the inner 27 rings are F1:A2-positive or F29:B10-positive isolates. (B) pMNCRE44_6 (F1:A2:B20 [accession no. CP010882]) used as a reference compared to draft assemblies of ColIa-positive isolates. The outer 21 rings are F1:A2-positive isolates, and the inner 18 rings are F1:A2-positive or F29:B10-positive isolates. (C) pJJ1886-5 (F2:A1 [accession no. CP006789]) used as a reference compared to draft assemblies of all F2:A1-positive isolates. (D) pMNCRE44_6 (F1:A2:B20 [accession no. CP010882]) used as a reference compared to draft assemblies of all F2:A1-positive isolates. The outer two rings are coding regions corresponding to coordinates in GenBank files. The inner two rings represent G+C content and G+C skew, respectively. Asterisks at the zero coordinates designate plasmids of the same replicon allele combination for each respective reference plasmid.

To further confirm the presence or absence of these plasmid lineages within the broader collection of ST131 strains, whole-plasmid BLAST nucleotide similarity searches were performed, and the results are displayed in Fig. 6. Results support the finding that F29:B10 plasmids are distinct from F1:A2 plasmids (Fig. 6A and B) and that the F2:A1 plasmids typical of *H*30Rx and basal sublineages are highly conserved and distinct from the F1:A2 and F29:B10 ColIa-containing plasmids typical of *H*30R1 and non-*H*30R, respectively (Fig. 6C and D).

### IS*26* actively shapes the microevolution of currently circulating ST131 plasmids.

He et al. recently demonstrated that IS*26* plays a major role in plasmid evolution through replicative transposition and cointegrate formation (24). A hallmark of these transposition events is 8-bp target site duplications that are “tracers” of IS*26*-mediated evolution. Since the ST131 plasmids examined here had high copy numbers of IS*26*, we performed an analysis of the 8-bp flanking sequences of intact IS*26* elements (see Table S1 in the supplemental material).

10.1128/mSphere.00121-16.4 Table S1  IS*26-*flanking 8-bp sequences in sequenced ST131 plasmids. Shared, colored backgrounds represent shared flanking sequences; boxed sequences represent those found across plasmids. Flanking sequences without shaded backgrounds are unique in this data set. Download Table S1, DOCX file, 0.2 MB. Copyright © 2016 Johnson et al. 2016 Johnson et al. This content is distributed under the terms of the Creative Commons Attribution 4.0 International license.

Similar to the findings of He et al., among more than 50 copies of IS*26* examined, we found no left- and right-flanking 8-bp sequences that were identical within an individual IS*26* copy (24). Rather, we found that different copies of IS*26* within a given plasmid often had identical left- or right-flanking 8-bp sequences, some of which were conserved between different plasmids. This strongly indicates that rather than intermolecular transposition, the primary driver of IS*26*-mediated evolution of these plasmids is through (i) *trans* intramolecular transposition resulting in IS*26* duplication and segment inversion or (ii) *cis* intramolecular transposition resulting in deletion of plasmid segments. This was evident when comparing closely related plasmids (red numbering on Fig. 3 and 5). There was clear evidence of differentiation between plasmid types (F2:A1, F1:A2:B20, and F29:B10) based upon IS*26* flanking 8-bp sequences, with each plasmid allelic type having a distinct subset of flanking sequences that were shared between plasmids of the same type. For example, identical 8-bp flanking sequences were found for F1:A2:B20 plasmids pZH063_1 (*H*35), pG199_1 (*H*30S), and pSaT040 (*H*22) (Fig. 5). Similar 8-bp flanking sequences were also found surrounding island 1 in F2:A1 plasmids (Fig. 3), supporting the common origin of these plasmids.

We also found a logical progression in IS*26* 8-bp flanking sequences from basal to recent isolates, supporting initial acquisition of IS*26* elements followed by extensive intramolecular transposition leading to the current ST131-*H*30Rx strains (Fig. 3). For example, in pCD306 (*H*30S, basal to *H*30Rx), Islands 1 and 2 are downstream of the aerobactin and Sit iron acquisition-related systems, with the FIB replicon positioned between aerobactin-Sit and island 2-island 1. In pJJ1897, aerobactin-Sit is absent and the FIB-island 2-island 1 region is inverted yet retains IS*26* 8-bp sequences flanking island 1, which were also present in pCD306. This orientation is then conserved in all sequenced *H*30Rx plasmids, except that FIB is absent and instead is replaced by a conserved cluster of hypothetical genes separated from island 2 by an IS*1* copy, supporting the common origin for these plasmids from a basal strain (i.e., *H*22) within the ST131 clade.

Also, multiple IS*26* insertions were found in the F transfer region of sequenced *H*30Rx plasmids, exemplified in simplest form in pJJ1886-5 as an insertion between *traI* and *traH*. This initial insertion likely led to additional IS*26*-mediated rearrangements in plasmid pJJ2434, for example, where the transfer region was disrupted: *finO*-*traX*-*traI* and the RepFIIA replicon were translocated to between islands 1 and 2, a portion of the transfer region was deleted, and the remainder of the transfer region and its adjacent antibiotic resistance island were inverted in the original location. In *H*30Rx-origin plasmids pEC958 and uk_P46212, a different IS*26* insertion in the F transfer region apparently led to the addition of *bla*_TEM-1_ within the transfer region. These are only a few examples of the numerous rearrangements and deletions that seemingly have occurred during the evolution of this plasmid within the *H*30Rx clade, particularly within antibiotic resistance-associated islands. Given the compressed time frame of *H*30Rx clade evolution (6), it is evident that IS*26*-mediated plasmid rearrangements are ongoing in the *H*30Rx clade.

### ST131 F-type plasmids have a comparatively low *in vitro* transfer rate.

Sequence evidence suggests that the plasmids of *H*30R1 and *H*30Rx may be fixed within their lineage, rarely transferring between strains. Using representative plasmids and host strains (see Table S2 in the supplemental material), we assessed the transfer frequency of ST131-source F2:A1 and F1:A2:B20 plasmids into several host backgrounds, including laboratory strains DH10B and K-12 MG1655, and (ST131) *H*22 to *H*30R1 strains lacking plasmids (see Fig. S2 in the supplemental material). Strains containing typical FIIA/FIB, I1, and χ^2^ plasmids were included for comparison as donors representing typical non-ST131 plasmids of different replicon types.

10.1128/mSphere.00121-16.2 Figure S2  Transfer frequencies of various plasmid types into different host backgrounds. Results are displayed as the log_10_ inverse number of transconjugants per donor, with increasing values indicating reduced transfer frequency. Results of three replicates per combination are shown. The lower limit of detectable conjugal frequency was 1 × 10^−8^ transconjugants/donor. Download Figure S2, TIF file, 0.2 MB. Copyright © 2016 Johnson et al. 2016 Johnson et al. This content is distributed under the terms of the Creative Commons Attribution 4.0 International license.

10.1128/mSphere.00121-16.5 Table S2  Strains used for plasmid transfer experiments. Download Table S2, DOCX file, 0.1 MB. Copyright © 2016 Johnson et al. 2016 Johnson et al. This content is distributed under the terms of the Creative Commons Attribution 4.0 International license.

The transfer frequencies of ST131-source plasmids were much lower than those of non-ST131-source plasmids (see Fig. S2 in the supplemental material). In particular, the transfer frequencies of ST131-source plasmids were 2 to 3 log_10_ lower than a prototypic F-type ColV plasmid from strain APEC O2 (22, 25). The tested *H*30R1 plasmids transferred at higher frequency than the tested *H*30Rx plasmids, possibly due to the innate transfer properties of F2:A1 versus F1:A2:B20 plasmids or due to a helper effect from the additional plasmids harbored by the tested *H*30R1 strains tested acting as helpers in conjugation (see Table S2 in the supplemental material). Different host backgrounds did not significantly influence the transfer frequencies of ST131-*H*30Rx plasmids (see Fig. S2). Overall, there appears to be a generalized deficiency in the ability of ST131-*H*30Rx plasmids to transfer spontaneously into recipient hosts that is independent of donor-recipient matching and that likely is due to the observed disruptions of the F transfer region in sequenced *H*30Rx plasmids.

### ST131-*H*30Rx plasmids demonstrate differential *in vitro* stability.

Sequence data also suggested that some strains within the *H*30R1 and *H*30Rx sublineages have lost their F-type plasmids, even though such plasmids are thought to be highly stable in the *E. coli* host (8). The *in vitro* stability of the plasmids of strains JJ1886 and JJ1887 was tested in LB broth during daily subculture over 14 days (approximately 140 generations) (26). Strains JJ1886 and JJ1887 are very closely related strains from two sisters, one of whom had recurrent cystitis (strain JJ1887), the other of whom developed fatal urosepsis (strain JJ1886) after caring for her sister with cystitis (13). The strains share four plasmids (pJJ1886-1, pJJ1886-2, pJJ1886-3, and pJJ1886-5). After 140 generations, JJ1886 retained its five plasmids in 100% of the population, while 33% of the JJ1887 population, on average, had lost plasmid pJJ1887-4 (the equivalent of pJJ1886-5 in strain JJ1886). Although this was a short experiment in the context of experimental evolution, it demonstrates possible differences in plasmid stability even between closely related ST131-*H*30Rx strains. This may explain why some ST131-*H*30Rx strains have lost their F2:A1 plasmids. That is, during microevolution within the clade, mutational events in the chromosome and/or plasmid may create differences in plasmid stability between even closely related strains.

### The fitness cost of plasmid carriage is diminished in *H*30R1 strains compared with ancestral *H*30 and laboratory strains.

Some have postulated that the fitness cost for carriage of ST131 plasmids may be reduced due to epistatic interactions between plasmids and specific host strain backgrounds (27). To test this, strain competitions were performed between common reference strain K-12 MG1655 and several ST131 wild-type strains and their plasmid-containing derivatives (see Fig. S3 in the supplemental material). Using a mean competitive index as a metric, we observed two key findings. First, competitive indices in LB broth demonstrated decreasing strain fitness by sublineage, ranging from (most fit) *H*30S > *H*30Rx > *H*30R1 (least fit). When the plasmids of strain JJ1886 (*H*30Rx) were introduced into different host backgrounds, competitive indices demonstrated a decreasing cost of plasmid carriage from (greatest cost) K-12 MG1655 > *H*30S > *H*30R1 (least cost). In fact, no significant cost of plasmid carriage was observed in the *H*30R1 genetic background. This indicates that even though overall fitness is reduced for *H*30R1 and *H*30Rx compared to *H*30S, adaptive and epistatic changes may have occurred during the transition from basal clades to the *H*30R1/Rx genetic background, resulting in overall lower fitness costs for carriage of these plasmids and thus favoring plasmid retention. Further work is necessary to determine which specific traits or mutations enable these possible epistatic interactions.

10.1128/mSphere.00121-16.3 Figure S3  The fitness cost of plasmid carriage is diminished in *H*30R1 strains compared with ancestral *H*30 and laboratory strains. Over 2 days in LB broth, *E. coli* strain K-12 was competed as a common reference against representative ST131 strains and ST131 transconjugants containing pJJ1886-5. Results are displayed as proportions of the test strain to reference K-12 strain at time zero divided by the same proportions at the end time (2 days). Download Figure S3, TIF file, 0.1 MB. Copyright © 2016 Johnson et al. 2016 Johnson et al. This content is distributed under the terms of the Creative Commons Attribution 4.0 International license.

### What events led to the current F plasmid distributions in ST131?

**FIG 7:**
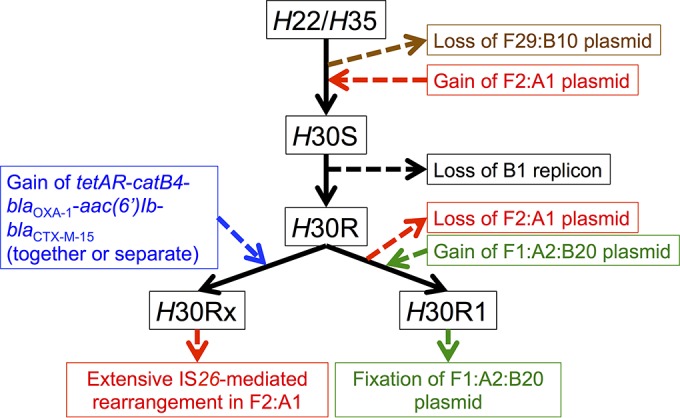
Proposed events leading to currently circulating plasmids in ST131-*H*30R1 and *H*30Rx.

Given the evidence provided here, we propose a sequence of events leading to the current circulating plasmids within the ST131-*H*30Rx sublineage (Fig. 7). A first key event was the introduction of an F2:A1 plasmid type into either the *H*22 or *H*30S sublineage basal to *H*30R1 and *H*30Rx. This plasmid may or may not have contained island 1, island 2, or aerobactin-Sit. It likely did not yet contain *bla*_CTX-M-15_, as highly similar versions of this plasmid that lacks this gene are present in basal isolates. Ancestral ST131 clade members likely contained different F plasmid types with overlapping traits (such as islands 1 and 2 and the ColIa-containing region), including the F29:B10 plasmid type found in basal *H*22 to *H*35 isolates. These ancestral plasmids likely were lost before the introduction of the F2:A1 plasmid into a precursor to *H*30R. Other possibilities certainly exist. It is possible islands 1 and 2 and/or aerobactin-Sit was introduced into the F2:A1 plasmid after its introduction into the ST131 clade from other plasmid types. It is also possible that plasmid or gene transfer between clades (from recent to basal or vice versa) has confounded the inferences presented here. However, the proposed events summarized in Fig. 7 are the most parsimonious, based on our analyses.

Following introduction of the F2:A1 plasmid containing islands 1 and 2 into *H*22/*H*30S, our data suggest that the B1 allele of the FIB replicon was subsequently lost. The *H*30R clade then formed, serving as the hypothetical ancestor to *H*30R1 and *H*30Rx. Based on our data, it is likely that an antibiotic resistance cassette containing *bla*_CTX-M-15_ and possibly also *catB4*, *bla*_OXA-1_, *aac(6′)Ib-cr*, and *tetAR*, was introduced via one or more IS*26*-mediated events. Along with accompanying chromosomal changes, this formed the *H*30Rx clade, which subsequently has been shaped extensively by IS-mediated acquisitions, deletions, and rearrangements on the plasmid. In some *H*30Rx strains, this resistance cassette was partially lost. In others, *bla*_CTX-M-15_ integrated into the bacterial chromosome. Also, some strains have lost the plasmid entirely or key portions of it.

If we assume, based on the data, that *H*30R1 and *H*30Rx have arisen in parallel from a common *H*30R ancestor and that the F2:A1 plasmid was present prior to (and, presumably, also in) this *H*30R hypothetical ancestor, this requires that an intermediate ancestor along the pathway from *H*30R to *H*30R1 must have lost the F2:A1 plasmid, subsequently acquiring the F1:A2:B20 plasmid found in current *H*30R1 strains. Of course, because of the challenges of interpreting sequence data from highly plastic elements, it is difficult to say to what extent recombination between plasmids impacted current plasmid formations. Given that all of the F replicon alleles were found, in some form, in *H*41 and early *H*22/*H*35 isolates, it is certainly possible that some of the current plasmids in *H*30R1 and *H*30Rx arose through extensive recombination of different F-type replicons. Our data are limited in this context, as we do not have completed plasmid sequences of all possible allelic combinations.

In summary, although considerable uncertainty remains regarding precisely how the ST131 clade evolved to contain currently circulating plasmids and resistance-associated genes, this study provides some key insights. First, ST131 contains several distinct plasmid types that were acquired independently. These plasmids’ evolutionary history has been challenging to interpret because they contain overlapping genetic traits, including typical F plasmid core genes and accessory islands such as islands 1 to 2 and the ColIa-containing region. However, phylogenetic inference supports the independent acquisition of these traits on multiple occasions, which fits with the concept of convergent evolution toward maintaining beneficial traits on highly plastic elements. It appears both that the *H*30R1/C1 and *H*30Rx/C2 clades may have coadapted with these plasmids to carry them at lower cost and that the plasmids themselves are evolving toward fixation within these clades through mutational events. Given the prominence of these plasmids among ST131 sublineages, it is likely that they play multiple roles in the success of their hosts.

## MATERIALS AND METHODS

### Bacterial strains.

A previous collection of 104 diverse ST131 strains described by Price et al. was used here (5). These included strains belonging to the following *fimH* allele-based subgroups: *H*22 (*n* = 26), *H*30S (*n* = 5), *H*30R1 (*n* = 32), *H*30Rx (*n* = 27), *H*35 (*n* = 7), and *H*41 (*n* = 4). Draft assemblies of all isolates from this collection were screened for plasmid-associated replicons and genetic loci, as described below. From this collection, 10 strains, including at least one representative from each of the major ST131 *fimH* allele-based subgroups included in the collection, were selected for additional PacBio sequencing (Table 1). Several additional (non-ST131) reference plasmids were also used for comparative analyses (Table 1).

### DNA sequencing.

Nine strains were sequenced using PacBio technology at the Rochester Mayo Medical Genome Facility (Rochester, MN). SMRTbell template libraries were generated from previously isolated unsheared raw genomic DNA using the Pacific Biosciences SMRTbell template prep kit 1.0 (Pacific Biosciences, Menlo Park, CA). Finished DNA libraries were subsequently subjected to DNA size selection using the BluePippin DNA size selection system (Sage Science, Inc.), with a 7-kb cutoff to select DNA fragments greater than 7 kb. Sequencing was performed on the PacBio RSII (Pacific Biosciences) using P6 polymerase binding and C4 sequencing kits, with magnetic bead loading and 240-min acquisition.

### Analysis and annotation.

Genome assemblies from PacBio data were created using HGAP 3 as part of SMRTAnalysis version 2.2. Assemblies were subjected to three rounds of polishing with Quiver. Completed and circular chromosomes or plasmids were reoriented to their replicon (for plasmids) or the origin of replication (for chromosomes) and subjected to a final round of polishing with Quiver. Genome annotation was performed using the National Center for Biotechnology Information (NCBI) Prokaryotic Genomes Automatic Annotation Pipeline (PGAAP; http://www.ncbi.nlm.nih.gov/genome/annotation_prok/) followed by manual curation for F-type plasmids. XPlasMap was used to construct plasmid maps (http://www.iayork.com/Widgets.shtml#XPlasMap). Resistance genes were identified using the CARD antibiotic resistance database (28). Plasmid types were identified using PlasmidFinder (29). F plasmid sequence types were identified using pMLST (29).

### Plasmid genome comparisons.

Comparisons of the completed sequences of ST131 plasmids with the existing collection of 104 ST131 draft genome assemblies were performed using the CGView comparison tool (30). Similarities between plasmid genomes were calculated using nucleotide BLAST with a 1,000-bp sliding window and an E value of 1 × 10^−6^. Circular comparison maps were then generated. Completed plasmids and reference sequences were aligned using MAUVE (31). Core single nucleotide variants from this alignment were extracted from nonrepetitive regions and analyzed using maximum parsimony methods with 1,000 bootstrap replicates in MEGA5 (32).

### Plasmid profiling.

Isolation and visualization of plasmids from the 10 isolates sequenced via PacBio were performed using pulsed-field gel electrophoresis (PFGE) with S1 nuclease (33), as previously described (12), following the procedures outlined by the Centers for Disease Control and Prevention PulseNet protocols (http://www.cdc.gov/pulsenet/pdf/ecoli-shigella-salmonella-pfge-protocol-508c.pdf). Strain JJ1886 was used as a positive control and size standard as it contains four plasmids ranging in size from 5 to 110 kb (13).

### Plasmid transfer experiments.

Conjugal transfer of plasmids from eight donor strains into five recipient strains representing different genetic backgrounds was performed using liquid mating experiments (see Table S2 in the supplemental material). Donor and recipient strains were grown overnight in 2 ml LB broth without antibiotic selection. The next day, the donor strain was inoculated 1:100 in fresh LB broth and allowed to grow for 3 to 4 h. Donor and recipient cells were mixed at volumes of 0.2 and 1.8 ml, respectively, and incubated at 37°C without shaking for 18 h. Eighteen hours was chosen for incubation after standard pilot experiments at 3 and 6 h failed to yield transconjugants from ST131 donors (data not shown). Following incubation, cultures were vortexed heavily, serially diluted, and plated in 100-µl aliquots onto MacConkey agar containing ampicillin (100 µg/ml, for donor plasmid selection) with and without rifampin (100 µg/ml, for recipient background selection). Conjugation frequencies were expressed as transconjugants per donor cell. Three replicates were performed for each strain combination.

### *In vitro* competitions.

Using *E. coli* K-12 strain MG1655 as a common reference, competitions were performed in LB broth against representative strains of *H*30S (G199), *H*30R1 (MVAST0038), and *H*30Rx (JJ1886). Also included were transconjugant derivatives of the *H*30S and *H*30R1 strains containing plasmid pJJ1886-5, a prototype *H*30Rx plasmid from strain JJ1886. These transconjugant strains were generated by overnight matings between *E. coli* DH10B containing pJJ1886-5 as a donor strain and respective recipient strains, followed by selection on MacConkey agar containing nalidixic acid (30 µg/ml) and ampicillin (100 µg/ml) to identify lactose-positive transconjugant colonies. Plasmid transfer was verified using S1 nuclease PFGE, as previously described (33).

Competitions were subsequently performed by first growing strains individually overnight in 2 ml LB broth with appropriate antibiotic selection and then washing the overnight bacterial pellet twice in sterile phosphate-buffered saline. Twenty-five microliters of each competing strain was combined into 5 ml fresh LB broth. Ten-fold serial dilutions of this mixture were plated on appropriate selective media to obtain time zero counts for each strain. The mixture was then grown overnight with shaking at 37°C. The next day, 5 µl of the overnight growth was reinoculated into 5-ml fresh LB broth and again grown overnight. Serial dilutions and plating on selective media were performed from this second overnight growth to obtain final (48-h) counts of each strain. Competitive indices for each strain competed with *E. coli* K-12 strain MG1655 were calculated as the proportion of test to reference strain counts at time zero divided by the ratio of test to reference strain counts at 2 days (34).

### Plasmid stability experiments.

The stability of plasmids from two closely related *H*30Rx strains, JJ1886 and JJ1887, was tested via daily passage in LB broth for 14 days following a previously described protocol (26). Briefly, overnight cultures of strains were inoculated into fresh LB broth and grown overnight at 37C with shaking at 200 rpm. Each day, 5 µl of overnight culture was transferred into 5 ml fresh LB broth. At days 0, 7, and 14, samples were taken from the growth, serially diluted, and plated onto LB agar without antibiotics. A PCR assay targeting genes of the plasmids of JJ1886 and JJ1887 was developed (see Table S3 in the supplemental material). PCR was performed on 90 colonies from each replicate and time point to determine the prevalence of plasmids within the culture. An ST131-specific gene was also included in the assay to rule out contamination (35). Two replicates per strain were tested.

10.1128/mSphere.00121-16.6 Table S3  Primers and product sizes used for JJ1886 plasmid screening. Download Table S3, DOCX file, 0.1 MB. Copyright © 2016 Johnson et al. 2016 Johnson et al. This content is distributed under the terms of the Creative Commons Attribution 4.0 International license.

### Accession number(s).

Completed or draft genomes have been deposited in NCBI GenBank (Table 1). PacBio raw data are available in the NCBI SRA database under BioProject no. PRJNA307507.
