# Supplementary figures and images for: Separate F-Type Plasmids Have Shaped the Evolution of the H30 Subclone of Escherichia coli Sequence Type 131
Source: mSphere. 2016 Jun 29;1(4):e00121-16. doi: 10.1128/mSphere.00121-16 (PMC4933990; doi:10.1128/mSphere.00121-16)

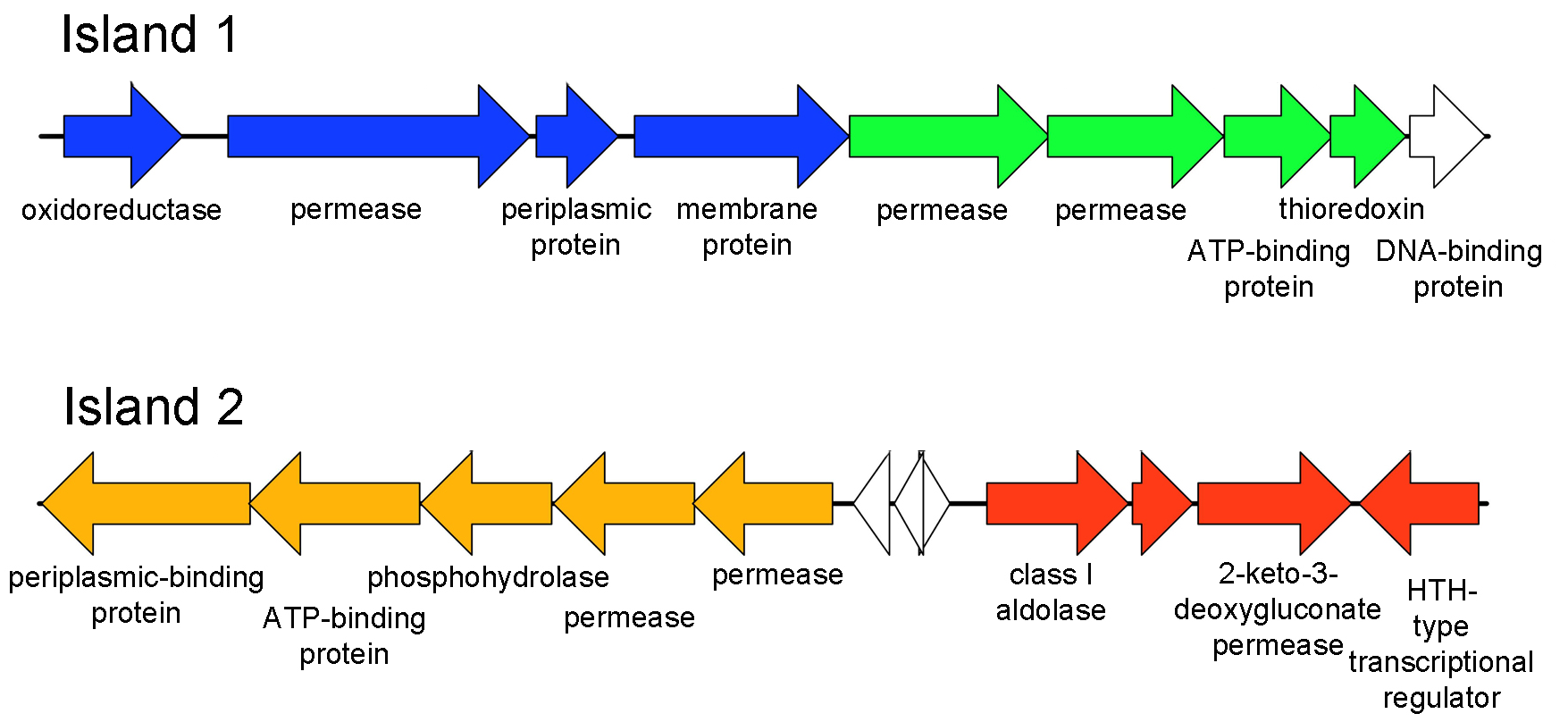

Supplement: Figure S1 [file sph004162108sf1.tif]

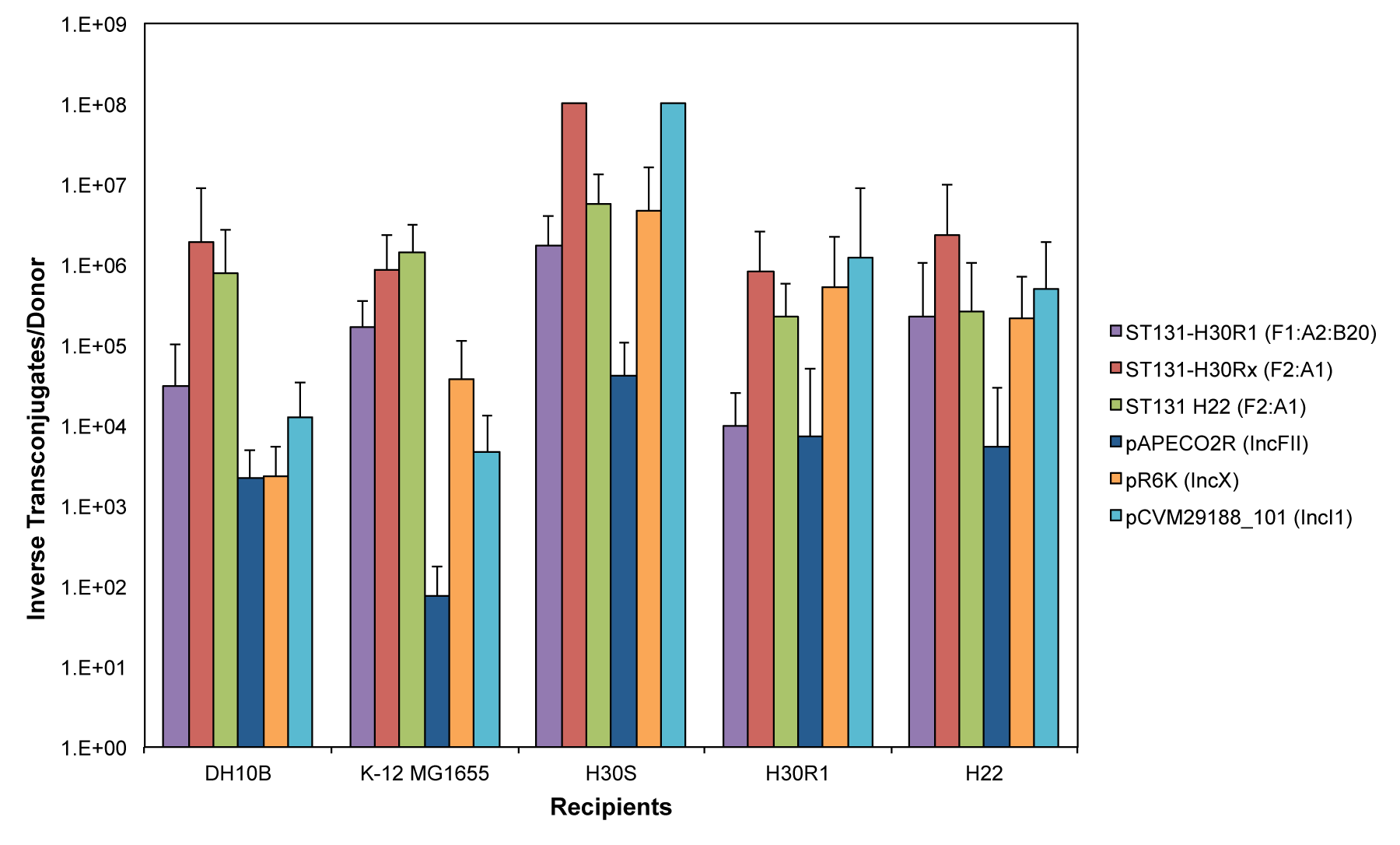

Supplement: Figure S2 [file sph004162108sf2.tif]

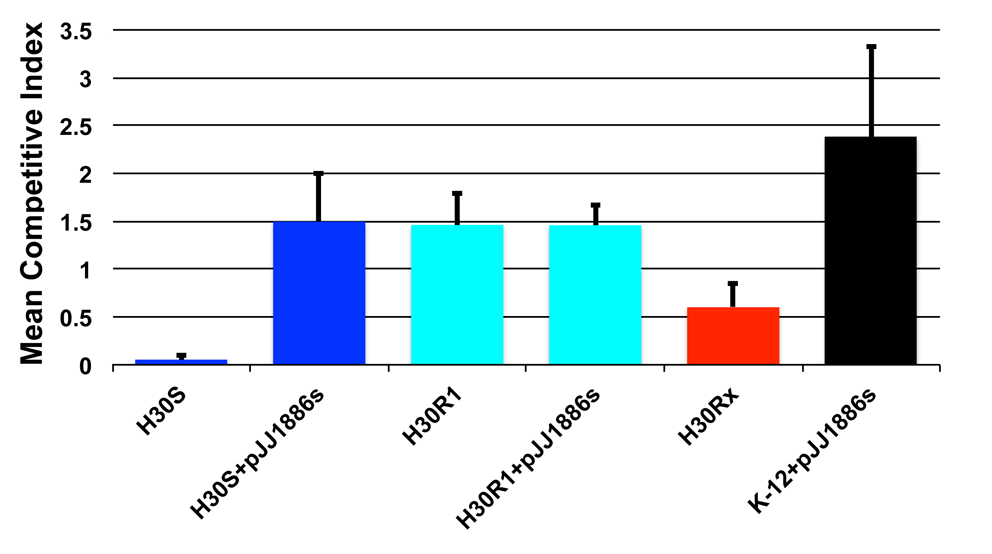

Supplement: Figure S3 [file sph004162108sf3.tif]
