# Supplementary material for: Separate F-Type Plasmids Have Shaped the Evolution of the H30 Subclone of Escherichia coli Sequence Type 131
Source: mSphere. 2016 Jun 29;1(4):e00121-16. doi: 10.1128/mSphere.00121-16 (PMC4933990; doi:10.1128/mSphere.00121-16)
Supplement: Table S1 [file sph004162108st4.docx]

Table S1. IS*26-*flanking 8-bp sequences in sequenced ST131 plasmids. Shared, colored backgrounds represent shared flanking sequences; boxed sequences represent those found across plasmids. Flanking sequences without shaded backgrounds are unique in this dataset. ­

| Lineage | Molecule | Start | Stop | Flank left pattern | Flank right pattern | Flank left | Flank right |
| --- | --- | --- | --- | --- | --- | --- | --- |
| H30S | pCD306 | 17913 | 17094 | 1 | 3 | AAAAATGC | TTCTACGG |
| H30S | pCD306 | 28740 | 27921 | 2 | 4 | CTTTCCAG | TCGTCAAG |
| H30R1 | pMNCRE44_6 | 67349 | 66530 | 5 | 6 | GGCAATAC | GTCGCAAC |
| H30R1 | pMNCRE44_6 | 76154 | 76973 | 6 | 7 | GTCGCAAC | GGTAATAG |
| H30R1 | pMNCRE44_6 | 119820 | 119003 | 7 | 28 | GGTAATAG | CGCGAGGT |
| H30Rx | pJJ1886_5 | 25080 | 25899 | 9 | 29 | ATTCTGAC | CGTGAAAT |
| H30Rx | pJJ1886_5 | 31187 | 32006 | 30 | 10 | GTTGAGCG | CCACATCT |
| H30Rx | pJJ1886_5 | 35012 | 34193 | 31 | 11 | AAGCTATG | TTTAAGCG |
| H30Rx | pJJ1886_5 | 48661 | 47882 | 32 |  | TCCTCCGG | AACAGAAC |
| H30Rx | pJJ1886_5 | 70931 | 70112 | 1 | 9 | AAAAATGC | ATTCTGAC |
| H30Rx | pJJ1886_5 | 81758 | 80939 | 2 | 4 | CTTTCCAG | TCGTCAAG |
| H22 | pJJ1897_1 | 37519 | 36700 | 33 | 12 | TCAGTAAG | GTCGCCGG |
| H22 | pJJ1897_1 | 39655 | 40474 | 34 | 3 | CTGCTGCC | TTCTACGG |
| H22 | pJJ1897_1 | 52798 | 51979 | 1 | 13 | AAAAATGC | GGATTGAA |
| H22 | pJJ1897_1 | 63624 | 62805 | 2 | 4 | CTTTCCAG | TCGTCAAG |
| H30Rx | pJJ2434_1 | 67532 | 68351 | 14 | 11 | TGTAGAAC | TTTAAGCG |
| H30Rx | pJJ2434_1 | 71357 | 70538 | 35 | 10 | GTCGAAGT | CCACATCT |
| H30Rx | pJJ2434_1 | 78975 | 78156 | 1 | 14 | AAAAATGC | TGTAGAAC |
| H30Rx | pJJ2434_1 | 58742 | 59561 | 15 | 37 | CGGTGACT | CGTTCCGT |
| H30Rx | pJJ2434_1 | 89802 | 88983 | 36 | 4 | TCAGAAGT | TCGTCAAG |
| H30Rx | pJJ2434_1 | 100559 | 99740 | 2 | 15 | CTTTCCAG | CGGTGACT |
| H41 | pMVAST0167_1 | 107988 | 107169 | 5 | 13 | GGCAATAC | GGATTGAA |
| H41 | pMVAST0167_1 | 97204 | 96385 | 38 | 16 | CGTGCGTC | CGAACAGA |
| H30R1 | pG150 | 116408 | 115589 | 39 | 40 | TCTATGGA | GGATTGAA |
| H30R1 | pG150 | 114526 | 113707 | 41 | 16 | CGCGCTTC | CGAACAGA |
| H30R1 | pG150 | 123883 | 123064 | 42 | 43 | ATCTAAAG | TACGTTCT |
| H30S | pG199_1 | 48278 | 49097 | 17 | 18 | GTTTTTTA | TAGTACAA |
| H22 | SaT040 | 84558 | 85377 | 17 | 18 | GTTTTTTA | TAGTACAA |
| H41 | ZH063 | 67688 | 68506 | 17 | 18 | GTTTTTTA | TAGTACAA |
| H30Rx | EC958 | 36289 | 37108 | 25 | 17 | GAAATACA | GTTTTTTA |
| H30Rx | EC958 | 44254 | 43435 | 26 | 27 | GTTACGTT | CGAGGCTG |
| H30Rx | EC958 | 75317 | 76136 | 44 | 4 | CGCAGAAA | TCGTCAAG |
| H30Rx | EC958 | 86144 | 86963 | 1 | 12 | AAAAATGC | GTCGCCGG |
| H30Rx | EC958 | 98864 | 98045 | 17 | 13 | GTTTTTTA | GGATTGAA |
| H30Rx | EC958 | 102450 | 103269 | 45 | 10 | AAAAATAG | CCACATCT |
| H30Rx | EC958 | 106275 | 105456 | 19 | 11 | GTCGAAGT | TTTAAGCG |
| H30Rx | EC958 | 114506 | 113687 | 46 | 47 | TTCCGACA | CGCAGAAA |
| NA^A^ | pRSB225 | 76655 | 77474 | 20 | 21 | CATCAGAG | CTTGATAT |
| NA | pRSB225 | 82680 | 81861 | 21 | 22 | CTTGATAT | GGTAAATC |
| NA | pRSB225 | 84597 | 85416 | 23 | 13 | ATATCGGC | GGATTGAA |
| NA | pRSB225 | 74795 | 75614 | 24 | 25 | CGCCGATG | AATTTATG |
| NA | pRSB225 | 116507 | 115688 | 2 | 4 | CTTTCCAG | TCGTCAAG |
| H30Rx | uk_P46212 | 6257 | 5438 | 1 | 13 | AAAAATGC | GGATTGAA |
| H30Rx | uk_P46212 | 83456 | 84275 | 25 | 17 | GAAATACA | GTTTTTTA |
| H30Rx | uk_P46212 | 129887 | 130706 | 19 | 11 | GTCGAAGT | TTTAAGCG |
| H30Rx | uk_P46212 | 133712 | 132893 | 48 | 10 | AATCAGAT | CCACATCT |
| H30Rx | uk_P46212 | 137285 | 138104 | 17 | 12 | GTTTTTTA | GTCGCCGG |
| H30Rx | uk_P46212 | 17084 | 16265 | 2 | 4 | CTTTCCAG | TCGTCAAG |
| H30Rx | uk_P46212 | 91421 | 90602 | 26 | 27 | GTTACGTT | CGAGGCTG |

^A^NA = not applicable, no associated host strain.
