# Supplementary material for: Separate F-Type Plasmids Have Shaped the Evolution of the H30 Subclone of Escherichia coli Sequence Type 131
Source: mSphere. 2016 Jun 29;1(4):e00121-16. doi: 10.1128/mSphere.00121-16 (PMC4933990; doi:10.1128/mSphere.00121-16)
Supplement: Table S2 [file sph004162108st5.docx]

**Table S2**. Strains used for plasmid transfer experiments.

| **Role** | **Origin** | **Strain** | **Plasmid(s)** | **Selective antibiotic** |
| --- | --- | --- | --- | --- |
| Donor | Wild type (*H*30Rx) | JJ1886 | pJJ1886-1, pJJ1886-2, pJJ1886-3, pJJ1886-4 (RepP), pJJ1886-5 (RepFIA/FIIA)^A^ | Ampicillin (100 ug/mL) |
| Donor | Wild type (*H*30Rx) | JJ1887 | pJJ1887-1, pJJ1887-2, pJJ1887-3, pJJ1887-4 (RepFIA/FIIA)^A^, pJJ1887-5 (RepFIB) | Ampicillin (100 ug/mL) |
| Donor | Wild type (*H*30Rx) | JJ2434 | pJJ2434-1 (RepFIA/FIIA)^A^, pJJ2434-2 | Ampicillin (100 ug/mL) |
| Donor | Wild type (*H*30R1) | MNCRE44 | pMNCRE44_1, pMNCRE44_2, pMNCRE44_3, pMNCRE44_4, pMNCRE44_5 (RepFIA/X3)^A^, pMNCRE44_6 (RepFIA/FIIA) | Ampicillin (100 ug/mL) |
| Donor | Wild type (*H*22) | JJ1897 | pJJ1897-1 (RepFIA/FIIA)^A^ | Ampicillin (100 ug/mL) |
| Donor | Wild type | APEC O2 | pAPEC-O2-R (RepFIIA)^A^, pAPEC-O2-ColV (RepFIIA/FIB) | Ampicillin (100 ug/mL) |
| Donor | Laboratory | K-12-Nal | pR6K (RepX2)^A^ | Ampicillin (100 ug/mL) |
| Donor | Laboratory | K-12-Nal | pCVM29188_101 (RepI1)^A^ | Ampicillin (100 ug/mL) |
| Recipient | Laboratory | DH10B | None | Rifampicin (100 ug/mL) |
| Recipient | Laboratory | K-12-Rif | None | Rifampicin (100 ug/mL) |
| Recipient | Wild type (*H*22) | SaT142 | None | Rifampicin (100 ug/mL) |
| Recipient | Wild type (*H*30S) | MVAST0131 | None | Rifampicin (100 ug/mL) |
| Recipient | Wild type (*H*30R1) | MVAST0038 | None | Rifampicin (100 ug/mL) |

^A^Plasmid(s) containing selectable marker.
