# Supplementary material for: Separate F-Type Plasmids Have Shaped the Evolution of the H30 Subclone of Escherichia coli Sequence Type 131
Source: mSphere. 2016 Jun 29;1(4):e00121-16. doi: 10.1128/mSphere.00121-16 (PMC4933990; doi:10.1128/mSphere.00121-16)
Supplement: Table S3 [file sph004162108st6.docx]

**Table S3**. Primers and products sizes used for JJ1886 plasmid screening.

| Gene | Sequence | Fragment Size (bp) |
| --- | --- | --- |
| PJJ1886_1_rep_fw | 5’-aggcaaagaaaaacaccgaggat-3’ | 408 |
| PJJ1886_1_rep_rv | 5’-ggcgctgaatatcaccgtcttt-3’ |  |
| PJJ1886_2_rep_fw | 5’-tttcgccccatcccgtaatgt-3’ | 354 |
| PJJ1886_2_rep_rv | 5’-gggggctgcgattgactgg-3’ |  |
| PJJ1886_3_hypo_fw | 5’-actcccggtcgtggcaaacat-3’ | 298 |
| PJJ1886_3_hypo_rv | 5’-gacgggcgcggagttcg-3’ |  |
| PJJ1886_4_topo_fw | 5’-aagcgccagcgttggtgtct-3’ | 459 |
| PJJ1886_4_topo_rv | 5’-gataatgtgccgcgcaatgaga-3’ |  |
| PJJ1886_4_klcb_fw | 5’-ctttcggccctcatagcctctc-3’ | 496 |
| PJJ1886_4_klcb_rv | 5’-ggggccagcacggtgatt-3’ |  |
| PJJ1886_5_ABC2_fw | 5’-agccgccgaagtcgtcgtt -3’ | 546 |
| PJJ1886_5_ABC2_rv | 5’-cggcgctggcgaaagaga-3’ |  |
| PJJ1886_5_perm_fw | 5’-gcggcggacgagggaaata-3’ | 255 |
| PJJ1886_5_perm_rv | 5’-cggctgaccggccttcatc -3’ |  |
| PJJ1886_5_rep_fw | 5’-cgcacgggcacgttttattc-3’ | 598 |
| PJJ1886_5_rep_rv | 5’-aggtgccggaacgctgaagtt-3’ |  |
